# Transcriptomic Analysis of the Impact of the *tet*(X4) Gene on the Growth Characteristics and Antibiotic Resistance Phenotypes of *Escherichia coli* Isolated from Musk Deer

**DOI:** 10.3390/ani15243564

**Published:** 2025-12-11

**Authors:** Kaiwei Yang, Xi Wu, Bingcun Ma, Jianguo Cheng, Zengting Li, Yin Wang, Zexiao Yang, Xueping Yao, Yan Luo

**Affiliations:** 1 College of Veterinary Medicine, Sichuan Agricultural University, Chengdu 611130, China; 2 Sichuan Institute for Drug Control, Chengdu 611731, China; 3 Sichuan Institute of Musk Deer Breeding, Dujiangyan 611800, China

**Keywords:** antibiotic resistance, *Escherichia coli*, tigecycline, *tet*(X4) gene, transcriptomics

## Abstract

*Escherichia coli* (*E. coli*) is a ubiquitous opportunistic pathogen in nature and serves as an important reservoir for antibiotic resistance genes. The *tet*(X4) gene is a key determinant mediating tigecycline resistance. Although its core resistance mechanism, encoding a flavin-dependent monooxygenase, has been elucidated, the broader impact of the *tet*(X4) gene on the secondary regulatory networks of *E. coli* remains not fully understood. In recent years, multiple studies have indicated that the *tet*(X4) gene participates in pathways contributing to resistance to other antibiotics by regulating the expression of various genes. In this study, *E. coli tet*(X4) gene deletion and complementation strains were constructed to investigate the mechanisms by which the *tet*(X4) gene influences the growth characteristics and antibiotic resistance of *E. coli*. The minimum inhibitory concentrations (MICs) of 24 different antibiotics, as well as the degradation capacities of tetracycline and tigecycline, were determined for the wild-type, deletion, and complementation strains. In addition, a four-week starvation stress experiment was performed under both the presence and absence of sub-inhibitory concentrations of tigecycline, during which the bacterial growth curves, survival rates, and MIC variations were analyzed. Transcriptomic sequencing of the wild-type, deletion, and complementation strains identified 531 differentially expressed genes associated with ABC transporter activity, drug metabolism, and bacterial two-component systems. These findings provide reliable evidence for elucidating the mechanism by which the *tet*(X4) gene affects *E. coli* resistance, offering valuable insights into the prevention and control of tigecycline-resistant *E. coli* infections.

## Simple Summary

*Escherichia coli* (*E. coli*) is an opportunistic pathogen and an important reservoir of antibiotic resistance genes. The *tet*(X4) gene mediates tigecycline resistance by encoding a flavin-dependent monooxygenase. However, the mechanism by which this gene influences bacterial antibiotic resistance through regulating the expression of other genes is complex and not yet fully understood. In this study, *E. coli tet*(X4) deletion and complemented strains were constructed to explore the effects of this gene on bacterial growth and antibiotic resistance. We measured the minimum inhibitory concentrations (MICs) of 24 antibiotics and the degradation capability for tetracycline/tigecycline in the wild type, deletion, and complemented strains, and conducted a 4-week starvation stress experiment (with/without subinhibitory concentrations of tigecycline) to analyze growth curves, survival rates, and changes in MICs. Transcriptome sequencing identified 531 differentially expressed genes, which are associated with ABC transporter activity, drug metabolism, and two-component systems. These findings provide reliable insights into the mechanism by which the *tet*(X4) gene affects *E. coli* antibiotic resistance and offer a reference for the prevention and control of tigecycline-resistant *E. coli* infections.

## 1. Introduction

*Escherichia coli* (*E. coli*) is a widely distributed opportunistic pathogen in the natural environment and is regarded as an important reservoir of bacterial antibiotic resistance genes [1]. It can not only carry numerous resistance determinants but also transfer them to other bacterial species through various horizontal gene transfer mechanisms. Multidrug-resistant (MDR) *E. coli* strains can spread extensively via air, food, and water, causing serious diseases such as diarrhea, septicemia, meningitis, pericarditis, and peritonitis in both humans and animals [2,3,4]. *E. coli* is the most common cause of bloodstream infections in adults worldwide, the second most common cause of neonatal meningitis, and a leading cause of sepsis and hospital-acquired infections or deaths in the United States. In addition, *E. coli* is a major pathogen of urinary tract infections and one of the most common pathogens in hospital-acquired and healthcare-associated infections. The prevalence of MDR *E. coli* has been steadily increasing, and tigecycline-resistant *E. coli* isolates are being detected with growing frequency. These strains, which often harbor multiple resistance genes, can render many commonly used antibiotics—including those considered the “last line of defense”—ineffective [5,6,7]. Infections caused by multidrug-resistant *E. coli* are not only difficult to prevent and treat, but also pose serious threats to human health and the economic development of the livestock industry. Antibiotics are the main means of treating *E. coli* infections in animals, but the continuous development of intensive and large-scale farming practices has led to widespread global antibiotic misuse, resulting in increased bacterial resistance and a surge in the number of superbugs [8]. In certain regions, *E. coli* resistance rates to fluoroquinolones and other critical antibiotics have exceeded 50%. This not only complicates treatment, leading to increased hospitalization and healthcare burdens, but also results in higher medical costs and increased mortality rates.

With the introduction of the “One Health” concept, bacterial antimicrobial resistance has gained increasing attention as a critical global public health issue [9]. In recent years, the growing resistance to carbapenems and colistin has left tigecycline as the last effective therapeutic option for treating MDR bacterial infections. The World Health Organization (WHO) has classified tigecycline as a critically important antimicrobial that should be used under strict regulation. Tigecycline acts by reversibly binding to the *16S* rRNA within the *30S* ribosomal subunit of bacteria, thereby preventing tRNA from entering the A site and inhibiting the transcription–translation process of protein synthesis [10]. Although tigecycline can overcome two major resistance mechanisms—ribosomal protection and active efflux—the *tet*(X) gene family mediates tigecycline resistance through an enzymatic inactivation mechanism. In particular, the emergence and dissemination of the *tet*(X4) gene, which confers high-level tigecycline resistance, have posed a severe challenge to the prevention and control of MDR bacterial infections [11]. Although tigecycline use is prohibited in food-producing animals, the detection rate of *tet*(X) genes in animal-derived bacteria has increased dramatically in recent years. Studies suggest that this phenomenon is closely linked to the extensive, long-term global use of tetracycline antibiotics over the past several decades [12,13,14]. Persistent selective pressure may therefore represent the major driving force behind the emergence and spread of *tet*(X) genes. The mechanisms underlying tigecycline resistance in *E. coli* are highly complex, and existing research remains insufficient. Thus, in-depth investigation of *tet*(X4)-mediated tigecycline resistance mechanisms in *E. coli* is essential for improving strategies to prevent and control infections caused by MDR *E. coli*.

The forest musk deer (*Moschus berezovskii*), a mammal belonging to the order *Artiodactyla*, family *Moschidae*, and genus *Moschus*, is mainly distributed in Sichuan, Yunnan, and Shaanxi provinces of China. Due to excessive hunting, wild musk deer populations have declined drastically, and the species has been listed as a National Class I Protected Animal in China and as an endangered species on the IUCN Red List since 2015. The mature male musk deer produces musk in its inguinal gland, which serves as a precious traditional Chinese medicinal ingredient and a high-value natural fragrance. To meet the demand for musk while protecting wild populations, artificial breeding programs for forest musk deer have been established. However, *E. coli* infection remains one of the most common diseases in captive musk deer, and with the escalating problem of bacterial resistance, these infections are becoming an increasingly serious threat to the health and survival of captive animals [15,16,17].

In this study, an multidrug-resistant *E. coli* strain carrying the *tet*(X4) resistance gene was isolated from a forest musk deer on a farm located in Sichuan Province, China. By constructing *E. coli tet*(X4) gene deletion and complementation mutants, we investigated the role of the *tet*(X4) gene in bacterial resistance phenotypes and growth characteristics. Furthermore, transcriptomic sequencing was employed to analyze differential gene expression between the wild-type and deletion strains under sub-inhibitory concentrations of tigecycline. This work provides new insights into the mechanisms of tigecycline resistance in *E. coli* and offers a theoretical basis for the prevention and control of tigecycline-resistant *E. coli* infections, as well as for the identification of potential novel antimicrobial targets.

## 2. Materials and Methods

### 2.1. Strains, Plasmids, and Culture Conditions

The bacterial strains and plasmids used in this study are listed in Supplementary Table S1. Strains were grown on Luria–Bertani (LB) agar plates (Haibo, Qingdao, China). Strains harboring the modified plasmid pRE112 were cultured in LB medium supplemented with chloramphenicol (Aladdin, Shanghai, China) (50 µg/mL) and polymyxin B (Aladdin, Shanghai, China) (2 µg/mL). *Escherichia coli* WM3064 was cultured in LB medium containing 2,6-diaminopimelic acid (DAP) (Aladdin, Shanghai, China) at a final concentration of 50 µg/mL. Strains carrying plasmid pBAD18 were induced to express the *tet*(X4) gene throughout the growth process by adding L-arabinose at a final concentration of 100 µg/mL. All bacterial strains were incubated at 37 °C.

### 2.2. Construction of the ΔtetX Deletion Mutant and the ΔtetX::tetX Complemented Strain

Following the method described by Zi and Li et al. [18,19], a *tet*(X4) gene deletion mutant was constructed using the suicide vector pRE112 and a double-crossover allelic exchange technique. Briefly, the upstream and downstream homologous arms of the *tet*(X4) gene were amplified using primer pairs UP-F/R and DOWN-F/R listed in Supplementary Table S2. The amplified fragments were fused by overlap PCR and subsequently ligated into the suicide plasmid pRE112 to generate the recombinant plasmid pRE112-ΔtetX. This recombinant plasmid was sequentially introduced into *E. coli* SM10-λpir and *E. coli* WM3064.Through conjugative transfer, the recombinant suicide plasmid was mobilized from the donor strain WM3064 into the recipient strain SCHYC01, where it was integrated into the chromosome via homologous recombination. The *sacB* gene was then activated by culturing the recombinant cells on LB medium containing 10% sucrose, which induced counterselection and resulted in the excision of the suicide plasmid from the SCHYC01 genome. The obtained *tet*(X4) gene deletion mutant was designated as ΔtetX. For complementation, the wild-type *tet*(X4) gene was cloned into the expression vector pBAD18 and introduced into competent ΔtetX cells by electroporation. The resulting complemented strain was designated as ΔtetX::tetX.

### 2.3. Antibiotic Susceptibility Testing

Antibiotic susceptibility of strains SCHYC01, ΔtetX, and ΔtetX::tetX to 24 antibiotics was determined using the broth microdilution method in accordance with the guidelines and interpretive standards of the Clinical and Laboratory Standards Institute (CLSI). The antibiotics tested included piperacillin/tazobactam 10:1 (TZP), ampicillin/sulbactam 1:1 (SAM), amoxicillin/clavulanic acid 2:1 (AMC), ceftazidime (CAZ), cefuroxime sodium (CXM), ceftriaxone (CRO), cefazolin (KZ), cefoxitin (FOX), aztreonam (ATM), imipenem (IPM), meropenem (MEM), gentamicin (CN), tobramycin (TOB), amikacin (AK), trimethoprim-sulfamethoxazole (SXT), polymyxin B (PB), nitrofurantoin (NFT), levofloxacin (LEV), ciprofloxacin (CIP), florfenicol (FFC), chloramphenicol (CHL), tetracycline (TE), doxycycline (DO), and tigecycline (TGC). The breakpoint for tigecycline was determined according to the criteria established by the U.S. Food and Drug Administration (FDA). *Escherichia coli* ATCC 25922 was used as the quality control strain [20,21,22].

### 2.4. Bioassay for Tetracycline Inactivation Mediated by the tet(X4) Gene

The functional expression of Tet(X)-type monooxygenases can be verified by the appearance of a deep orange pigment in bacterial cultures incubated with tetracyclines. To assess the enzymatic activity of Tet(X4), the method described by Umar et al. [23] was followed. Bacterial cultures in the logarithmic growth phase were inoculated into 100 mL of LB medium containing tetracycline at a final concentration of 100 µg/mL and tigecycline at 8 µg/mL, and incubated at 37 °C with shaking at 180 r/min for 24 h. LB medium without bacterial inoculation served as the blank control, while bacterial cultures grown without tetracycline served as the negative control. For strains harboring the plasmid pBAD18, 0.1% L-arabinose was added to the culture medium to induce expression of the *tet*(X4) gene from the pBAD18 vector.

### 2.5. Tigecycline Degradation Assay

Following the method described by Chen et al. [24], strains SCHYC01, ΔtetX, ΔtetX::tetX, and ΔtetX–pBAD18 were cultured in LB broth containing tigecycline at sub-inhibitory concentrations specific to each strain. Cultures were incubated at 37 °C with shaking at 180 r/min for 12 h, after which the bacterial suspensions were centrifuged at 13,000 rpm for 20 min, and the supernatants were filtered through 0.22 μm filters (Thermo Fisher Scientific, Shanghai, China). 200 µL of an overnight culture of tigecycline-sensitive *E. coli* ATCC 25922 were spread on Mueller–Hinton (MH) agar plates (Haibo, Qingdao, China). A 6 mm diameter paper disk containing 2 µg of tigecycline was placed at the center of each plate. To observe inhibition zones, 20 µL of the previously obtained filtered supernatant were added dropwise onto the paper disk, and the plates were incubated at 37 °C for 16 h. Controls included plates with no supernatant added and plates with supernatant obtained from *E. coli* ΔtetX–pBAD18 (carrying the empty vector pBAD18).

### 2.6. Starvation Survival Assay

The starvation survival assay was conducted in two groups following the method described by Wan et al. [25]. Bacterial cultures in the mid-logarithmic phase were adjusted to a concentration of 1 × 10^8^ CFU/mL, centrifuged at 6000 rpm for 5 min to remove the supernatant, and the cell pellets were resuspended and homogenized in sterile phosphate-buffered saline (PBS) (Aladdin, Shanghai, China). This washing step was repeated three times to completely eliminate residual culture medium.

For the first group, the washed bacterial suspensions were incubated in sterile PBS at 37 °C with shaking at 180 r/min. For the second group, the cells were incubated under the same conditions in sterile PBS containing a sub-inhibitory concentration of tigecycline, which was replenished every 24 h. Bacterial counts were determined daily using the serial dilution and plate counting method. Survival rates were calculated by comparing the CFU of cultures at each time point with that of the initial population. After the 28-day experiment, the minimum inhibitory concentrations (MICs) of the strains were re-evaluated and compared with the pre-experimental data to assess any changes. Each experiment was performed in triplicate.

### 2.7. RNA Extraction and Transcriptomic Sequencing

Total RNA was extracted from strains SCHYC01, ΔtetX, and ΔtetX::tetX using the TRIzol method, followed by removal of genomic DNA. Ribosomal RNA was depleted using the RiboCop rRNA Depletion Kit for Mixed Bacterial Samples (Lexogen, Greenland, NH, USA), and mRNA was randomly fragmented into 200 nt segments by adding fragmentation buffer (Illumina^®^ Stranded mRNA Prep, Ligation, San Diego, CA, USA). First-strand cDNA synthesis was performed using random hexamer primers (Illumina), with dUTP incorporated during second-strand synthesis. The cDNA was then subjected to end-repair, phosphorylation, and ‘A’ base addition following the Illumina^®^ Stranded mRNA Prep protocol (San Diego, CA, USA). The resulting RNA-seq libraries were sequenced on the Illumina Novaseq6000 platform (Illumina Inc., San Diego, CA, USA) [26,27].

### 2.8. RT-qPCR Validation of Transcriptomic Sequencing Data

Reverse transcription quantitative PCR (RT-qPCR) was performed to validate the transcriptomic sequencing data. Strain SCHYC01, ΔtetX and ΔtetX::tetX cDNA synthesis was conducted using the Evo M-MLV RT Master Mix for qPCR kit (AG11706, Accurate Biology, Changsha, China), followed by quantitative PCR with SYBR Green Pro Taq HS Premix kit (AG11701, Accurate Biology, China) on a Bio-Rad CFX96 system. The thermal cycling protocol consisted of an initial denaturation at 95 °C for 30 s, followed by 40 cycles of 95 °C for 5 s and 60 °C for 30 s. Amplification specificity was verified by melting curve analysis. All reactions were performed in triplicate, and relative gene expression was quantified using the 2^−ΔΔCt^ method with *16S* rRNA as the internal control. Primer sequences are shown in Supplementary Table S2.

### 2.9. Statistical Analysis

Data were analyzed using GraphPad Prism 8.0 and SPSS 27.0, with results expressed as mean ± SD. Group comparisons were performed using *t*-tests and two-way ANOVA, with statistical significance defined as *p* < 0.05 and high significance as *p* < 0.01. All experiments were repeated independently in triplicate to ensure reproducibility.

## 3. Results

### 3.1. Construction and Verification of the tet(X4) Gene Deletion and Complemented Strains

**Figure 1:**
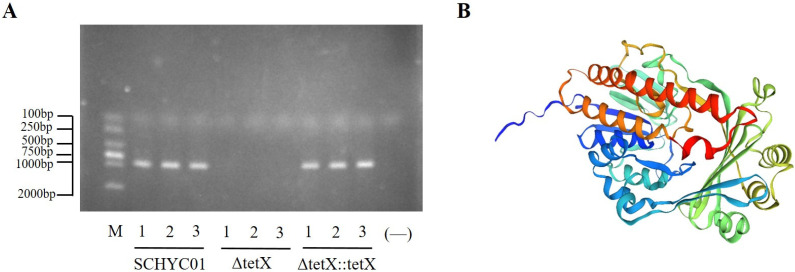
(**A**) Agarose gel electrophoresis analysis of PCR products amplified using *tet*(X4)-specific primers (tet-F/R). (**B**) Predicted three-dimensional protein structure of *tet*(X4) generated by SWISS-MODEL.

To confirm the successful construction of the ΔtetX and ΔtetX::tetX strains, genetic stability testing, PCR amplification, and RT-qPCR analysis were performed. As shown in Figure 1A, agarose gel electrophoresis results indicated that when DNA from the wild-type, deletion, and complemented strains was amplified using the tetX-F/R primer pair, no corresponding fragment was amplified in the deletion strain, while both the wild-type and complemented strains produced a band of 1134 bp. Sequencing analysis further verified that the amplified fragments matched the expected *tet*(X4) sequence. RT-qPCR results showed that no fluorescent signal corresponding to the *tet*(X4) gene was detected in the deletion strain. After 30 serial passages, both the deletion and complemented strains maintained genetic stability, with no evidence of *tet*(X4) gene loss, mutation, or recombination.

Genetic analysis of the *tet*(X4) gene carried by the SCHYC01 strain revealed that, similar to the typical arrangement of *tet*(X4), it is located within an approximately 3.1 kb fragment referred to as the ‘*tet*(X4)-containing box.’ Within this region, the *tet*(X4) gene is positioned downstream of the hydrolase gene *catD*. The 3′ and 5′ ends of the box containing *catD*-*tet*(X4) are linked to IS*CR2* sequences [28]. Figure 1B illustrates the predicted three-dimensional structure of Tet(X4) protein using SWISS-MODEL.

### 3.2. Determination of Antibiotic Susceptibility in Strains SCHYC01, ΔtetX, and ΔtetX::tetX

Table 1 presents the minimum inhibitory concentrations (MICs) and susceptibility profiles of strains SCHYC01, ΔtetX, and ΔtetX::tetX against 24 antibiotics. The results indicate that the primary differences in MIC values among the three strains were observed for tetracycline-class antibiotics, with the most pronounced difference occurring in tigecycline susceptibility. Additionally, the ΔtetX strain exhibited slight variations in MIC values for ceftazidime (CAZ), ceftriaxone (CRO), imipenem (IPM), tobramycin (TOB), polymyxin B (PB), and chloramphenicol (CHL) compared with the other two strains; however, changes in resistance classification were only detected for imipenem and tobramycin.

### 3.3. Determination of Growth Curves of Strains SCHYC01, ΔtetX, and ΔtetX::tetX

**Figure 2:**
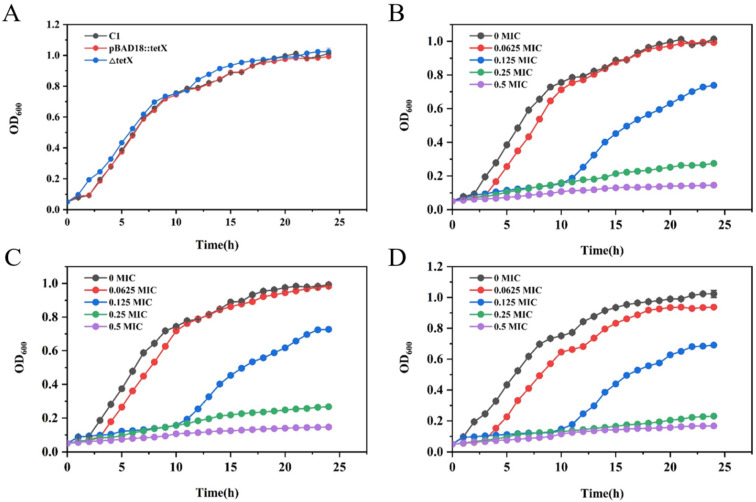
(**A**) Growth curves of strains SCHYC01, ΔtetX, and ΔtetX::tetX. (**B**) Growth curves of strain SCHYC01 under different concentrations of tigecycline pressure. (**C**) Growth curves of strain ΔtetX under different concentrations of tigecycline pressure. (**D**) Growth curves of ΔtetX::tetX under different concentrations of tigecycline pressure.

As shown in Figure 2A, the overall growth curves of strains SCHYC01, ΔtetX, and ΔtetX::tetX exhibited no significant differences under antibiotic-free conditions. The growth curves of SCHYC01 (Figure 2B), ΔtetX (Figure 2C), and ΔtetX::tetX (Figure 2D) were further determined over a 24 h period in the presence of tigecycline at concentrations corresponding to 0.5, 0.25, 0.125, and 0.0625 fold of each strain’s MIC value. The results showed that although bacterial growth rates were inhibited at 0.125 × MIC, the overall growth trends of all three strains were not significantly affected. Therefore, this concentration was selected as the sub-inhibitory tigecycline concentration for pretreatment prior to transcriptomic sequencing and for subsequent experiments, in order to better assess gene expression changes in response to tigecycline pressure.

### 3.4. Inactivation of Tetracycline-Class Antibiotics by Tet(X4)

**Figure 3:**
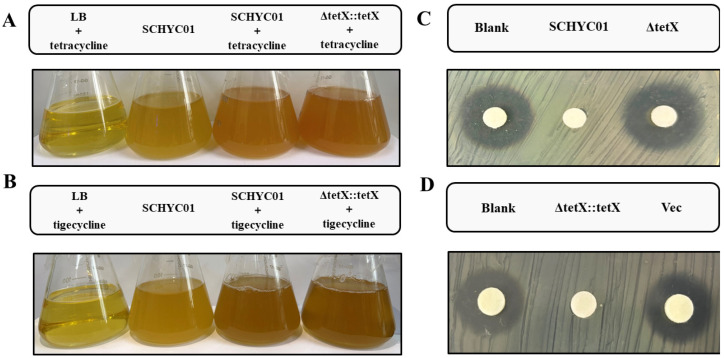
Detection of the inactivation effect of *E. coli* strains on tetracycline antibiotics. (**A**) The color of the medium after culturing strains SCHYC01 and ΔtetX::tetX in media containing different concentrations of tetracycline. (**B**) The color of the medium after culturing strains SCHYC01 and ΔtetX::tetX in media containing different concentrations of tigecycline. (**C**) The effect of supernatants from strains SCHYC01 and ΔtetX cultures on the tigecycline antibiotic susceptibility test inhibition zone diameter. (**D**) Effect of culture supernatants from strains ΔtetX::tetX and ΔtetX–pBAD18 on the inhibition zone diameter in tigecycline susceptibility tests.

As shown in Figure 3A, compared with bacterial cultures without tetracycline, the cultures of strains SCHYC01 and ΔtetX::tetX that were supplemented with tetracycline at a final concentration of 100 μg/mL developed a distinctly deeper orange color. Similarly, in another set of experiments using tigecycline at a final concentration of 8 μg/mL (Figure 3B), comparable color changes were observed. These results indicate that the monooxygenase encoded by the *tet*(X4) gene expressed in strains SCHYC01 and ΔtetX::tetX effectively catalyzes the degradation of tetracycline-class antibiotics.

To further verify the tigecycline-degrading activity of the monooxygenase encoded by the *tet*(X4) gene, 20 μL of filtered supernatants from overnight cultures of each strain were applied to tigecycline-containing antibiotic disks, as shown in Figure 3C,D. The supernatants from strains SCHYC01 and ΔtetX::tetX markedly reduced the diameter of the inhibition zones, whereas the supernatants from ΔtetX and ΔtetX–pBAD18 (carrying the empty vector) had no observable effect on inhibition zone size.

### 3.5. Starvation Survival Assay

**Figure 4:**
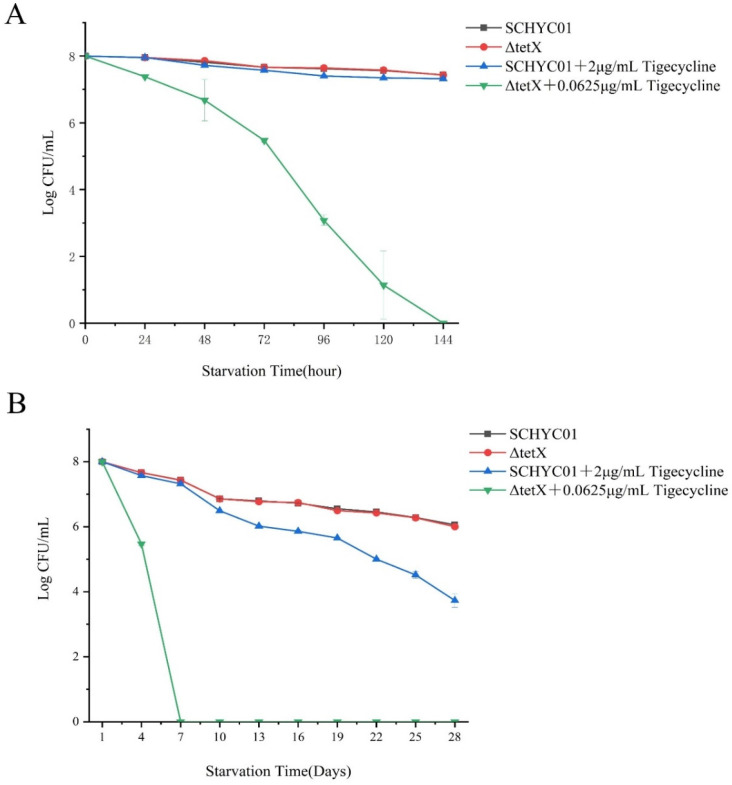
Survival levels of *E. coli* SCHYC01 and the gene deletion mutant ΔtetX under starvation stress. (**A**) Survival levels of the strains during the first 7 days under different stress conditions. (**B**) Survival levels of the strains during the first 28 days under different stress conditions.

As shown in Figure 4A, under starvation stress without the addition of sub-inhibitory concentrations of tigecycline, strains SCHYC01 and ΔtetX exhibited similar survival rates after seven days, both maintaining relatively high viability. However, when ΔtetX was exposed to starvation stress in the presence of sub-inhibitory tigecycline concentrations, its survival rate declined sharply, and complete cell death occurred after the seventh day. As shown in Figure 4B, in the absence of tigecycline, both SCHYC01 and ΔtetX maintained high survival rates under starvation conditions. Moreover, strain SCHYC01 retained substantial viability even under combined starvation stress and sub-inhibitory tigecycline exposure.

As presented in Table 2 and Table 3, the MIC values of tetracycline-class antibiotics for strain SCHYC01 showed no significant changes during the 28-day starvation stress experiment. However, under tigecycline pressure, the MIC value of SCHYC01 for tigecycline doubled after the third week, while its MIC value for tetracycline showed a slight decrease.

### 3.6. Effect of tet(X4) Gene Deletion on Global Regulatory Functions in E. coli and RT-qPCR Validation of Transcriptomic Data

**Figure 5:**
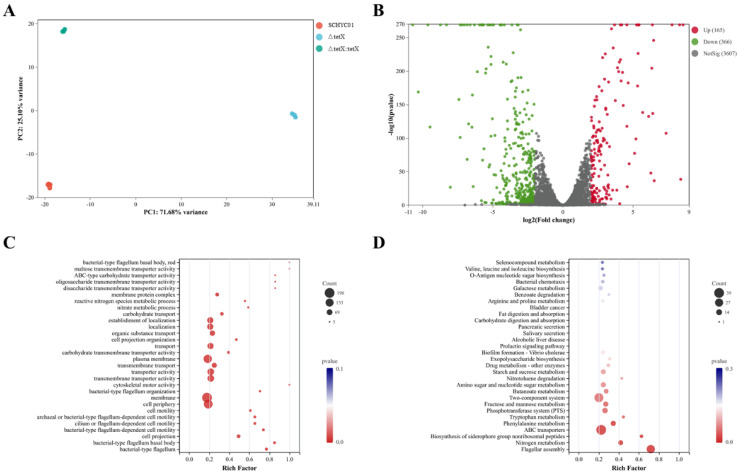
(**A**) Principal component analysis plot. (**B**) Scatter plot of DEGs between strain SCHYC01 and strain ΔtetX. (**C**) Bubble GO enrichment analysis chart showing rich factors versus GO term. (**D**) KEGG pathway enrichment map of DEGs.

Comprehensive transcriptomic analyses were performed on the three *E. coli* strains to investigate the impact of *tet*(X4) gene deletion on gene expression under sub-inhibitory tigecycline pressure. As shown in Figure 5, a total of 531 differentially expressed genes (DEGs) (|log_2_FC| ≥ 2, *p* < 0.05) were identified in the *tet*(X4) deletion mutant compared with the wild-type strain, including 165 upregulated and 366 downregulated genes. Relative expression levels of genes such as *rpoZ*, *ftnA*, *coaD*, *bfr*, and *RS08470* were significantly increased (*p* < 0.001), whereas genes including *punC*, *RS20315*, *RS14735*, *lam*B, and *RS09070* were significantly downregulated (*p* < 0.001) in the *tet*(X4) deletion mutant compared with the wild-type and complemented strains.

**Figure 6:**
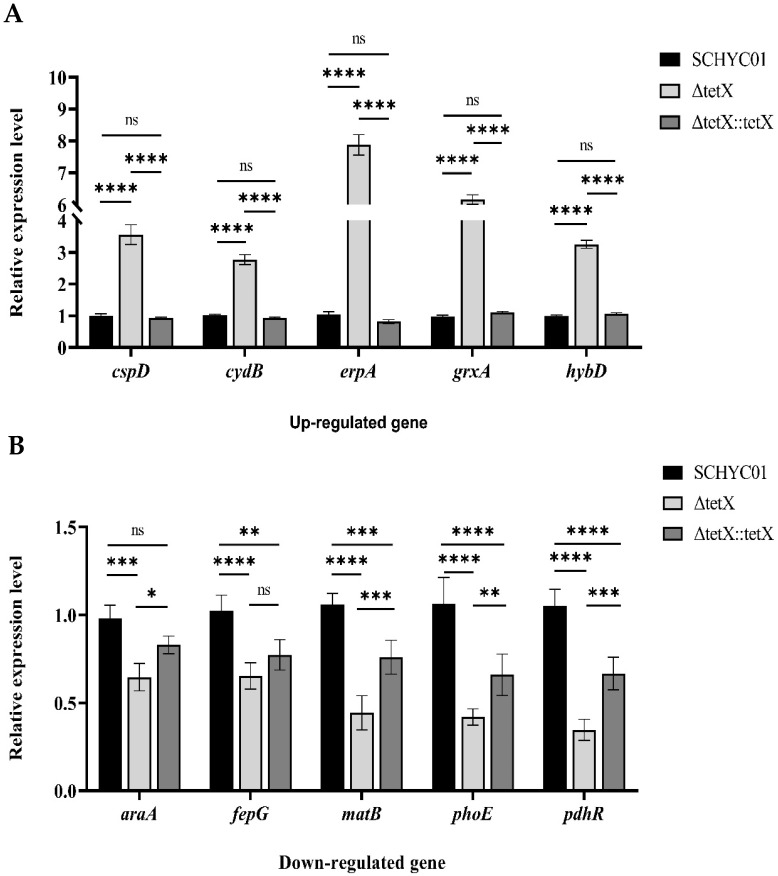
(**A**) RT-qPCR validation of five upregulated genes identified in transcriptomic analysis. (**B**) RT-qPCR validation of five downregulated genes identified in transcriptomic analysis. (Note: ns *p* > 0.05, * *p* < 0.05, ** *p* < 0.01, *** *p* < 0.001, **** *p* < 0.0001).

As shown in Figure 6, 5 upregulated and 5 downregulated DEGs were randomly selected for RT-qPCR analysis to validate the accuracy of the transcriptomic data. The RT-qPCR results were consistent with the transcriptome-based predictions, confirming the reliability of the sequencing data.

**Figure 7:**
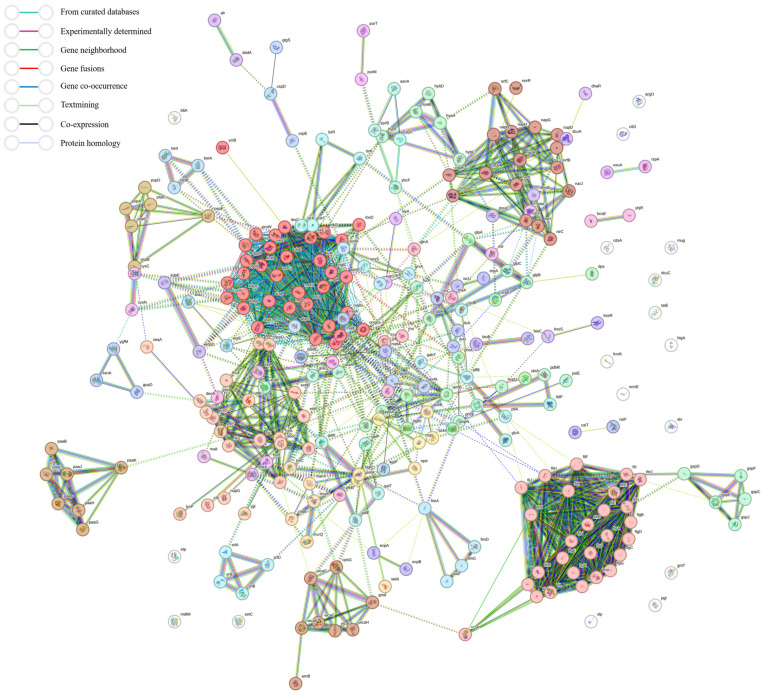
Differential gene interaction network diagram between strain SCHYC01 and strain ΔtetX. Note: STRING Version 12.0 is sourced from https://string-db.org/ (accessed on 10 October 2025).

Construct a protein–protein interaction (PPI) network for all differentially expressed genes (DEGs) using STRING version 12.0. This systematically demonstrates the functional associations among the 280 differentially expressed proteins present in the database. In the network, each circular node represents the tertiary structure of the encoded protein, and nodes with more similar colors indicate stronger functional correlations. In the interaction network, the greatest number of genes are associated with ABC transporters, followed by genes related to flagellar assembly, nitrate assimilation, Macrolide metabolism, and Siderophore transmembrane transport. The number of genes involved in each of these groups exceeds ten (Figure 7).

Functional enrichment analysis revealed that multiple pathways were significantly enriched under antibiotic stress. Gene Ontology (GO) enrichment analysis showed that DEGs were primarily associated with molecular functions such as transmembrane transporter activity, transporter function, and carbohydrate transmembrane transport activity. Furthermore, KEGG pathway enrichment analysis indicated that DEGs were mainly mapped to pathways related to xenobiotic biodegradation and metabolism, terpenoid and polyketide metabolism, ABC transporter–mediated membrane transport, glycoside biosynthesis and metabolism, and energy metabolism.

**Figure 8:**
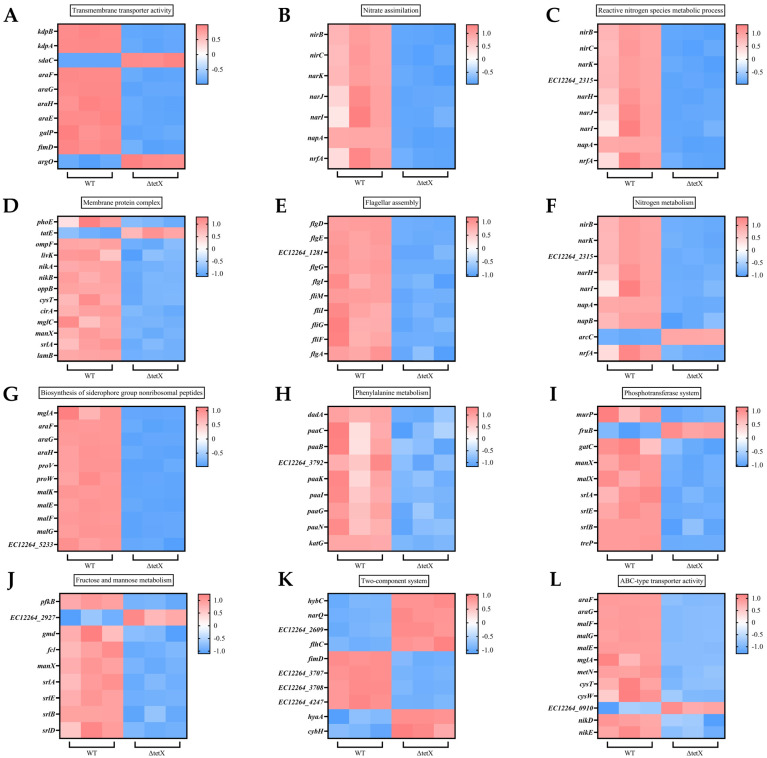
Partial gene expression levels of strains SCHYC01 and ΔtetX. (**A**) Transmembrane transporter activity. (**B**) Nitrate assimilation. (**C**) Reactive nitrogen species metabolic process. (**D**) Membrane protein complex. (**E**) Flagellar assembly. (**F**) Nitrogen metabolism. (**G**) Biosynthesis of siderophore group nonribosomal peptides. (**H**) Phenylalanine metabolism. (**I**) Phosphotransferase system. (**J**) Fructose and mannose metabolism. (**K**) Two-component system. (**L**) ABC-type transporter activity.

As shown in Figure 8, we compared the differential gene expression associated with Transmembrane transporter activity, Nitrate assimilation, Reactive nitrogen species metabolic process, and Membrane protein complex that were enriched in the GO analysis. In addition, we presented the differential expression profiles of genes enriched in KEGG pathways including Flagellar assembly, Nitrogen metabolism, Biosynthesis of siderophore group nonribosomal peptides, Phenylalanine metabolism, Phosphotransferase system, Fructose and mannose metabolism, Two-component system, and ABC-type transporter activity. Compared to the wild-type strain, the deletion strain showed significant downregulation of genes associated with transmembrane transporter activity, including *kdpB*, *araF*, *glaP*, and *fimD*; genes related to bacterial two-component systems, such as *EC12264_3707*, *EC12264_3708*, *EC12264_4247*, and *fimD*; and genes related to ABC-type transporter activity, such as *araF*, *malG*, *metN*, and *cysW*. This downregulation may be one of the reasons for the increased antibiotic sensitivity observed in the deletion strain.

## 4. Discussion

With the growing severity of bacterial antimicrobial resistance, the prevention and control of infections caused by multidrug-resistant (MDR) pathogens and the limitation of resistance gene dissemination have become issues of increasing concern in public health. Tigecycline, a broad-spectrum tetracycline antibiotic, plays a crucial role in inhibiting MDR bacteria [17]. It was first approved for clinical use in the United States in 2005 and introduced into clinical practice in China in 2011. According to the China Antimicrobial Surveillance Network (CHINET), as of 2019, only 3.5% of Enterobacteriaceae and 4.5% of carbapenem-resistant Enterobacteriaceae isolates in China exhibited resistance to tigecycline. Compared with polymyxins, which also demonstrate strong efficacy against MDR bacteria, tigecycline has the advantage of not inducing nephrotoxicity, providing a higher safety profile in clinical applications [29,30,31]. The *tet*(X4) gene, identified for the first time in 2019, is a key determinant mediating tigecycline resistance. It encodes an enzyme capable of degrading all tetracycline-class antibiotics, including tigecycline and eravacycline. The emergence of *tet*(X4) threatens the clinical effectiveness of tigecycline and has raised considerable concern within the medical and scientific communities [11].

In this study, the *Escherichia coli* (*E. coli*) strain SCHYC01 also carries the *tet*(X4) resistance gene, marking the first isolation of an *E. coli* strain with the *tet*(X4) gene from captive forest musk deer [32]. The MIC determination results showed that this strain exhibits high resistance to tigecycline. Some studies suggest that its ancestral form of the gene existed even before the use of tigecycline. *R. anatipestifer* has been reported as a probable source of the *tet*(X) gene. A tracking screen for *tet*(X) in public databases revealed that it appeared as early as the 1960s in *R. anatipestifer* and was the primary *tet*(X) vector during the initial stages. Comparative genomic analysis indicated that *tet*(X) variants were likely produced through the dissemination of *tet*(X) between *Flavobacteriaceae* and *E. coli*/*Acinetobacter* species, with IS*CR2* playing a pivotal role, leading to the hypothesis that *R. anatipestifer* might be a potential natural source of *tet*(X). This indicates that the *tet*(X4) gene has evolved through modifications such as point mutations and fragment recombination under the pressure of tetracycline antibiotics, particularly tigecycline [10,17,33,34]. Existing studies indicate that many tigecycline resistance genes are located near mobile genetic elements, which increases the risk of the spread of tigecycline resistance. The *tet*(X4) gene carried by the isolated strain SCHYC01 is flanked by the insertion sequence IS*CR2*, which has been shown to facilitate the spread of the *tet*(X4) gene on both bacterial chromosomes and plasmids [35,36,37]. The IS*CR2*-*tet*(X4)-*catD* combination, one of the most common and conserved mobile element structures, has been found in various host organisms. IS*CR2* belongs to the IS*91* family, which can transfer antimicrobial resistance genes via rolling-circle transposition. Unlike most insertion sequence elements, a single copy of IS*CR2* can transfer adjacent DNA sequences. Additionally, studies suggest that IS*CR2* may continue to capture other resistance genes during the transfer of the *tet*(X4) gene, progressively increasing the number of resistance genes it carries [38,39]. This can lead to the spread of more harmful resistance determinants. In the future, a deeper analysis of the interactions between tigecycline resistance genes and mobile genetic elements is essential. Understanding their mechanisms of spread will help mitigate the transmission of tigecycline resistance genes across different environments and species.

In this study, we also conducted a starvation stress experiment to evaluate the bacterial survival rates of the wild-type, deletion, and complemented strains under nutrient-deficient conditions and under sub-inhibitory tigecycline stress. The results showed that strain SCHYC01 maintained a high survival rate throughout the four-week starvation period. This strong survivability under extreme conditions enables pathogenic bacteria to persist in natural environments and withstand adverse external factors. When favorable growth conditions are restored, these bacteria can rapidly regain metabolic activity, resume normal physiological functions, and proliferate extensively. Moreover, the isolated strain demonstrated similarly high survival capacity under sub-inhibitory tigecycline stress. Such antibiotic tolerance is not only a key factor contributing to infection recurrence but is also associated with chronic bacterial infections, including tuberculosis, cystic fibrosis–related pulmonary infections, and candidiasis. The remarkable adaptive resilience of multidrug-resistant bacteria therefore poses an even greater threat to human and animal health [40]. However, this experiment did not assess the in vivo transmission efficiency, tissue colonization ability, or other biological characteristics of bacteria carrying the *tet*(X4) gene. As a result, it cannot fully replicate the complex host-microbe interactions in animals, such as immune clearance, microbial competition, and drug metabolism. In the future, we plan to conduct further experiments and attempt to develop relevant animal models to enrich the research data in this field.

In the transcriptomic sequencing analysis, enrichment of differentially expressed genes (DEGs) revealed significant involvement in pathways associated with bacterial two-component signal transduction systems (TCSs). TCSs are essential bacterial signaling mechanisms composed of a sensor histidine kinase and a response regulator. They play a crucial role in bacterial adaptation to environmental stresses such as temperature fluctuations, pH changes, osmotic pressure, and antibiotic exposure. Most bacteria possess several to dozens of TCSs, some of which can directly regulate the expression of antibiotic resistance genes, thereby enhancing bacterial resistance. For example, in Vibrio parahaemolyticus, the VbrKR system can sense the presence of β-lactam antibiotics and subsequently upregulate the expression of the blaA gene, which encodes a hydrolytic enzyme capable of degrading β-lactam antibiotics. Similarly [41], in *E. coli*, the EvgSA system regulates the expression of multiple drug efflux genes, including *emrKY*, *yhiUV*, *acrAB*, and *mdfA*, thereby conferring multidrug resistance (MDR) to the bacterium [42,43].

Among the various mechanisms contributing to bacterial antibiotic resistance, efflux pumps play a crucial role. By actively expelling antibiotics, disinfectants, and other toxic compounds from bacterial cells, efflux pumps reduce the intracellular concentrations of these agents, thereby facilitating resistance development and maintaining cellular metabolic stability. Consequently, bacterial efflux systems have become key targets in the development of novel antimicrobial agents [44]. In the present transcriptomic analysis, several differentially expressed genes were found to be associated with ABC-type transporter activity. Bacterial ATP-binding cassette (ABC) transporters consist of transmembrane domains (TMDs) and nucleotide-binding domains (NBDs). The TMDs contain substrate-binding pockets, while the NBDs bind and hydrolyze ATP to drive substrate translocation across the membrane [45]. ABC transporters exist in multiple forms. Among Gram-negative bacteria, the tripartite MacAB–TolC efflux pump is one of the most extensively studied. Previous studies have shown that deletion of the macAB efflux system increases bacterial susceptibility to aminoglycosides and polymyxins, whereas acquisition or overexpression of MacAB decreases antibiotic sensitivity [46,47,48]. Similarly, in Gram-positive bacteria, numerous ABC transporters have been implicated in antibiotic resistance. For instance, LmrA enhances the tolerance of Lactococcus lactis to macrolides and lincosamides; EfrAB in Enterococcus faecalis efficiently expels gentamicin, streptomycin, and chloramphenicol; and PatA/B in Streptococcus pneumoniae reduces susceptibility to fluoroquinolones such as ciprofloxacin and norfloxacin [49,50,51].

Based on the above analysis, we infer that strain SCHYC01 activates its intrinsic two-component system (TCS) under sub-inhibitory tigecycline pressure. The activated TCS may, in turn, enhance the efflux activity of the bacterial ABC transport system, thereby increasing the strain’s resistance to tigecycline. The regulatory interaction between bacterial two-component systems and efflux pump activity, which collectively influence antibiotic resistance, has been documented in previous studies. For example, it has been reported that the BaeSR TCS in Acinetobacter baumannii regulates the expression of *adeA* and *adeB* to cope with high extracellular antibiotic concentrations. In *E. coli*, deletion of the BasSR TCS results in loss of resistance to antibiotics such as norfloxacin, tetracycline, and erythromycin, accompanied by reduced expression of the multidrug efflux pump gene *emrD* [52]. Similarly, in the EvgA/EvgS TCS of *E. coli*, the sensor protein EvgS responds to environmental stimuli through its periplasmic domain and transduces the signal via a phosphorylation cascade to the response regulator EVGA, ultimately affecting the bacterium’s antibiotic susceptibility [53]. Efflux pumps in bacteria are finely regulated by multiple signaling networks, among which two-component systems play a particularly critical role. Given their widespread presence in bacterial species, TCSs represent promising new targets for the development of antimicrobial agents and for designing therapeutic strategies to combat infections caused by multidrug-resistant bacteria in animals.

In the analysis of the transcriptomic data of this study, we also found that the deletion strain of the *tet*(X4) gene exhibited downregulation of key genes of the type IV secretion system (T4SS), such as virB4 and traC. T4SS is a highly complex nanomachine that is crucial for bacteria to gain a competitive advantage, complete horizontal gene transfer, and mediate host–pathogen interactions [54,55]. T4SS consists of 12 protein components, VirB1-11 and VirD4, forming two major subassemblies: the outer membrane core complex (OMCC) and the inner membrane complex (IMC) [56]. Antibiotic resistance plasmids can spread resistance genes among bacteria through T4SS. Although T4SS is vital for conjugative transfer, this energy-intensive process imposes an adaptive cost on the host bacterium, and therefore the expression of T4SS is tightly regulated [57,58]. Recent studies have revealed that the transcriptional regulator TetR, encoded by the tetracycline resistance gene tetR carried on *Acinetobacter baumannii* resistance plasmids, can influence bacterial conjugation, physiological phenotypes, and pathogenicity through regulation of the type IV secretion system [59,60,61]. Currently, it remains unclear whether the *tet*(X4) gene in *E. coli* affects the expression of T4SS and the transmission of resistance plasmids mediated by it. In the future, elucidating the molecular mechanisms and structural basis by which the resistance gene *tet*(X4) modulates T4SS to influence conjugative transfer of resistance plasmids could provide new insights for the development of antibacterial drugs targeting multidrug-resistant *E. coli*.

## 5. Conclusions

According to our findings, deletion of the *tet*(X4) gene led to a marked reduction in the minimum inhibitory concentration (MIC) of tigecycline in *Escherichia coli* (*E. coli*) SCHYC01. Our study also confirmed that the *tet*(X4) resistance gene carried by strain SCHYC01 can directly inactivate both tetracycline and tigecycline, while simultaneously inducing widespread differential gene expression in the bacterium. The *tet*(X4) gene appears to activate bacterial two-component systems, which subsequently upregulate the activity of ABC transporters to help the bacteria adapt to tigecycline-induced stress. Our results lay the foundation for further exploration of the function of the *tet*(X4) gene and its role in bacterial adaptation and resistance under antibiotic stress. In the future, we will further explore the specific pathways enriched in transcriptome analysis and their related upstream and downstream regulatory mechanisms in order to contribute to the prevention and treatment of multidrug-resistant *E. coli* infections and the screening of novel antibacterial drug targets.

## Figures and Tables

**Table 1:** Antibiotic susceptibility test results of strains SCHYC01, ΔtetX, and ΔtetX::tetX.

Category of Antimicrobial	Antibiotic	Strain SCHYC01	Strain ΔtetX	Strain ΔtetX::tetX
β-lactams	piperacillin/tazobactam (TZP)	16/4 μg/mL (I)	16/4 μg/mL (I)	16/4 μg/mL (I)
	ampicillin/sulbactam (SAM)	32/16 μg/mL (R)	32/16 μg/mL (R)	32/16 μg/mL (R)
	amoxicillin/clavulanic acid (AMC)	32/16 μg/mL (R)	32/16 μg/mL (R)	32/16 μg/mL (R)
	ceftazidime (CAZ)	32 μg/mL (R)	16 μg/mL (R)	32 μg/mL (R)
	cefuroxime sodium (CXM)	32 μg/mL (R)	32 μg/mL (R)	32 μg/mL (R)
	ceftriaxone (CRO)	8 μg/mL (R)	4 μg/mL (R)	8 μg/mL (R)
	cephazolin (KZ)	16 μg/mL (R)	16 μg/mL (R)	16 μg/mL (R)
	cefoxitin (FOX)	32 μg/mL (R)	32 μg/mL (R)	32 μg/mL (R)
	aztreonam (ATM)	16 μg/mL (R)	16 μg/mL (R)	16 μg/mL (R)
	imipenem (IPM)	2 μg/mL (I)	1 μg/mL (S)	2 μg/mL (I)
	meropenem (MEM)	16 μg/mL (R)	8 μg/mL (R)	16 μg/mL (R)
aminoglycosides	gentamicin (CN)	16 μg/mL (R)	16 μg/mL (R)	16 μg/mL (R)
	tobramycin (TOB)	16 μg/mL (R)	8 μg/mL (I)	16 μg/mL (R)
	amikacin (AK)	64 μg/mL (R)	32 μg/mL (I)	64 μg/mL (R)
sulfonamides	trimethoprim-sulfamethoxazole (SXT)	1024 μg/mL (R)	1024 μg/mL (R)	1024 μg/mL (R)
polypeptides	polymyxin b (PB)	0.5 μg/mL (S)	0.25 μg/mL (S)	0.5 μg/mL (S)
furans	nitrofurantoin (NFT)	256 μg/mL (R)	128 μg/mL (R)	256 μg/mL (R)
quinolones	levofloxacin (LEV)	0.5 μg/mL (S)	0.5 μg/mL (S)	0.5 μg/mL (S)
	ciprofloxacin (CIP)	1 μg/mL (R)	1 μg/mL (R)	1 μg/mL (R)
amide alcohols	florfenicol (FFC)	32 μg/mL (R)	32 μg/mL (R)	32 μg/mL (R)
	chloramphenicol (CHL)	16 μg/mL (R)	8 μg/mL (I)	8 μg/mL (I)
tetracyclines	tetracycline (TE)	64 μg/mL (R)	16 μg/mL (R)	32 μg/mL (R)
	doxycycline (DO)	32 μg/mL (R)	16 μg/mL (R)	32 μg/mL (R)
	Tigecycline (TGC)	16 μg/mL (R)	0.25 μg/mL (S)	16 μg/mL (R)

Note: S: Sensitive; I: Intermediary; R: Resistance.

**Table 2:** Changes in the MIC of tetracycline antibiotics in strain SCHYC01 during starvation stress.

Days	Antibiotic (μg/mL)		
	Tetracycline	Doxycycline	Tigecycline
0	64	32	16
7	64	32	16
14	64	32	16
21	32	32	16
28	32	32	16

**Table 3:** Changes in the MIC of tetracycline antibiotics for strain SCHYC01 under tigecycline pressure during starvation stress.

Days	Antibiotic (μg/mL)		
	Tetracycline	Doxycycline	Tigecycline
0	64	32	16
7	64	32	16
14	32	32	16
21	32	32	32
28	32	32	32

## Data Availability

The original contributions presented in this study are included in the article/Supplementary Materials. Further inquiries can be directed to the corresponding author.

## Supplementary Materials

The following supporting information can be downloaded at: https://www.mdpi.com/article/10.3390/ani15243564/s1, Table S1: The bacterial strains and plasmids used in this study; Table S2: Primers used in this study.
